# Supplementary material for: HoloLens 1 vs. HoloLens 2: Improvements in the New Model for Orthopedic Oncological Interventions
Source: Sensors (Basel). 2022 Jun 29;22(13):4915. doi: 10.3390/s22134915 (PMC9269857; doi:10.3390/s22134915)

Supplementary material D2

Extra plots

Correlation plots between error and distance to origin

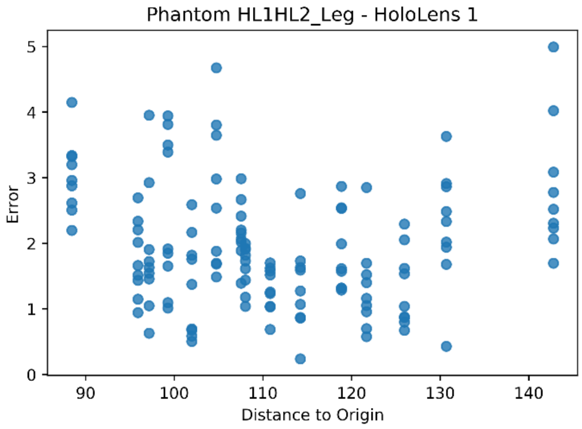

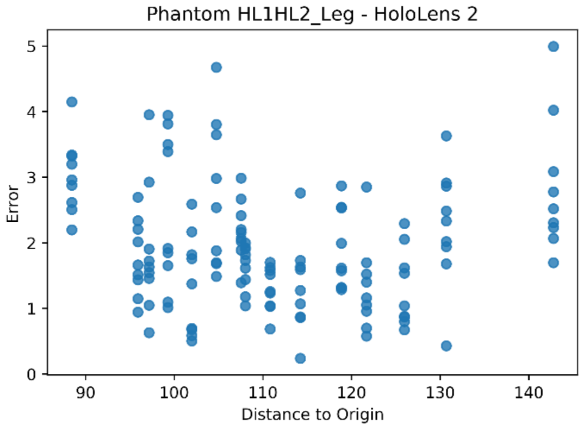

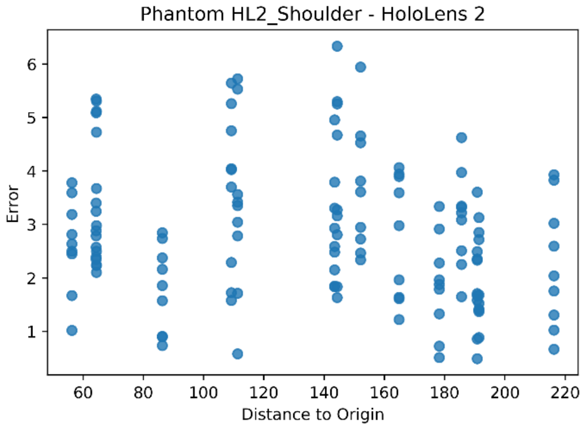

Connover matrices to analyze significant differences between the errors in each control point.

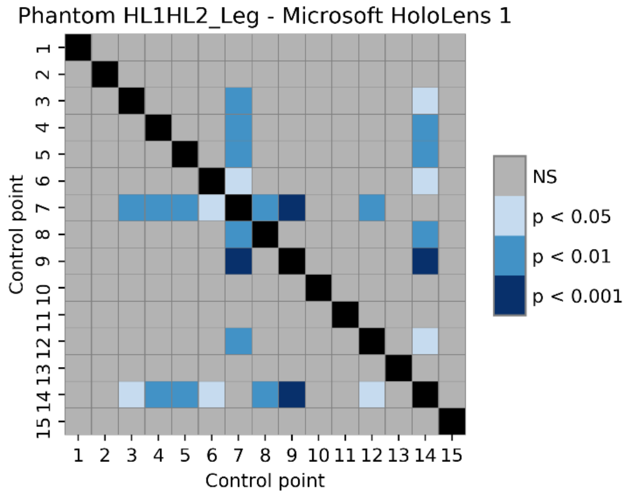

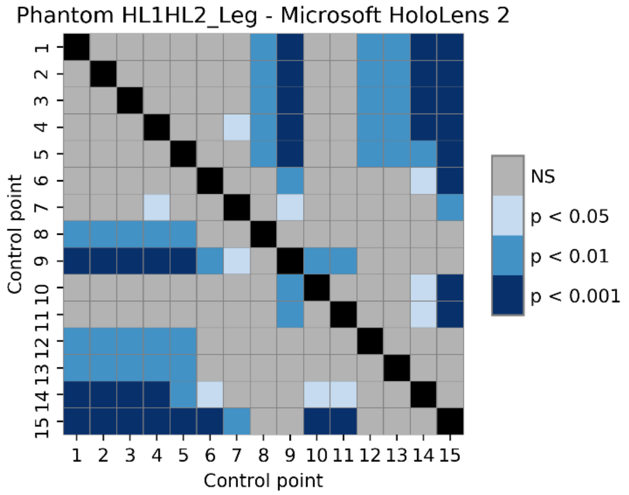

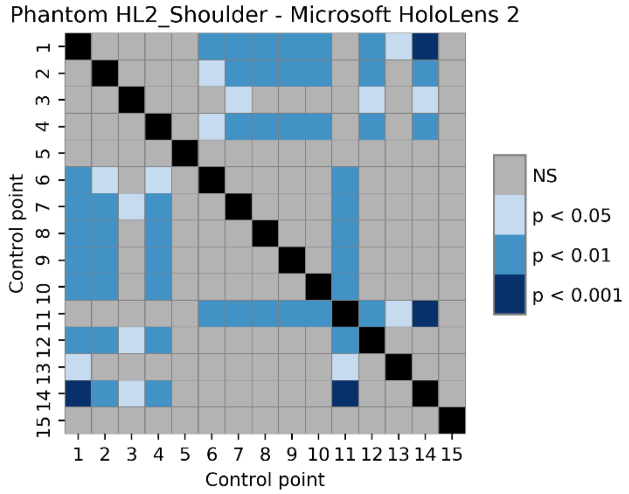

Supplement: Supplementary file 1 [file sensors-22-04915-s001.zip › SupplementaryMaterialDocument D2.pdf]
