# Supplementary material for: HoloLens 1 vs. HoloLens 2: Improvements in the New Model for Orthopedic Oncological Interventions
Source: Sensors (Basel). 2022 Jun 29;22(13):4915. doi: 10.3390/s22134915 (PMC9269857; doi:10.3390/s22134915)
Supplement: Supplementary file 1 [file sensors-22-04915-s001.zip › SupplementaryMaterial_Document D1.pdf]

Supplementary material D1

Survey to compare HoloLens 1 and HoloLens 2

Name	
Date	
Occupation	
Have you used both, HoloLens 1 and HoloLens 2, for clinical applications?	☐ Yes ☐ No

Question	Rating				
COMFORTABILITY AND WEIGHT					
1. Evaluate the HoloLens' adjustment and hold along time.	Very bad	-	-/+	+	Very good
2. Evaluate the comfortability (in general) of every device.	Very uncomfortable	-	-/+	+	Very comfortable
3. How would you consider the weight of the devices on the head?	Too heavy	-	-/+	+	Very light
HOLOGRAMS					
4. Have you ever experienced fatigue after wearing the glasses for long periods of time? If so, how much time were you wearing them?	I've never experienced fatigue	0-30 min	30-60 min	1-2 hours	> 2 hours
5. How would you rate the holograms' quality and their integration in the real world?	Not convincing at all	-	-/+	+	Very convincing
6. Interaction with holograms is intuitive/realistic.	Completely disagree	-	-/+	+	Completely agree
7. The devices response speed is high	Completely disagree	-	-/+	+	Completely agree
RECOGNITION					
8. How would you assess the HoloLens' hand recognition?	Terrible	-	-/+	+	Perfect
FIELD OF VIEW					
9. How do you consider the HoloLens' field of view is?	Terrible	-	-/+	+	Perfect
GENERIC QUESTIONS					
10. Would you include this technology in your rutinary workflow?	Yes			No	
11. Which device (HoloLens 1 or HoloLens 2) would you prefer?	HoloLens 1			HoloLens 2	
12. Which model do you think that could benefit the most the orthopedic oncological surgeries?	HoloLens 1			HoloLens 2	

Observations	
